# Modulation of the Vascular-Immune Environment in Metastatic Cancer

**DOI:** 10.3390/cancers13040810

**Published:** 2021-02-15

**Authors:** Bo He, Ruth Ganss

**Affiliations:** Cancer Microenvironment Laboratory, Harry Perkins Institute of Medical Research and the University of Western Australia Centre for Medical Research, The University of Western Australia, Nedlands, WA 6009, Australia; bo.he@perkins.org.au

**Keywords:** metastasis, immunotherapy, angiogenesis, tertiary lymphoid structures, vessel normalization, vessel co-option

## Abstract

**Simple Summary:**

Abnormal blood vessels restrict immune cell access into primary cancers and thus contribute to resistance to immunotherapy. In contrast, vessel remodeling or normalization strategies increase anti-cancer immune responses. Much less is known about the inter-relationship of blood vessels and immune cells at secondary metastatic sites. This review specifically explores how abnormal vascular features in the pre-metastatic niche can be targeted and potentially repaired. Moreover, the growth of established metastases relies on blood supply from both pre-existing, co-opted and newly formed angiogenic vessels. The co-existence of blood vessels with different maturation status may impact on vascular targeting strategies and responsiveness to immunotherapy, thus requiring innovative approaches to treat advanced cancer.

**Abstract:**

Advanced metastatic cancer is rarely curable. While immunotherapy has changed the oncological landscape profoundly, cure in metastatic disease remains the exception. Tumor blood vessels are crucial regulators of tumor perfusion, immune cell influx and metastatic dissemination. Indeed, vascular hyperpermeability is a key feature of primary tumors, the pre-metastatic niche in host tissue and overt metastases at secondary sites. Combining anti-angiogenesis and immune therapies may therefore unlock synergistic effects by inducing a stabilized vascular network permissive for effector T cell trafficking and function. However, anti-angiogenesis therapies, as currently applied, are hampered by intrinsic or adaptive resistance mechanisms at primary and distant tumor sites. In particular, heterogeneous vascular and immune environments which can arise in metastatic lesions of the same individual pose significant challenges for currently approved drugs. Thus, more consideration needs to be given to tailoring new combinations of vascular and immunotherapies, including dosage and timing regimens to specific disease microenvironments.

## 1. Introduction

Cancer mortality increases with metastatic tumor spread to distant organs, and advanced metastatic cancer is usually very challenging. Primary tumors develop in an environment of excessive angiogenesis, hypoxia and immune-suppressive inflammation which fosters growth and intravasation of metastatic cancer cells into the circulation. Similarly, microenvironmental changes in the pre-metastatic niche precede metastasis formation at distant sites [1]. Much like primary tumors, overt metastases thrive in a facilitative microenvironment. Thus, major current anti-cancer treatments such as anti-angiogenesis or immune therapies have shifted the focus from the cancer cells to the cancer microenvironment. Moreover, recent findings demonstrate an intricate regulatory network between the angiogenic vasculature and intra-tumoral innate and adaptive immune cells which could be harnessed to design more effective therapies [2,3]. However, there is considerable uncertainty about the composition of the vascular and immune microenvironment in different tumor stages [4]. Some therapies might be effective in the primary tumor environment and reduce metastatic tumor dissemination, but ineffective in preventing already circulating tumor cells from lodging and colonizing distant organs. Indeed, there is emerging evidence that the same treatment can have different outcomes when applied as neoadjuvant (before surgical removal of the primary tumor) or adjuvant (after surgery) therapy. For instance, anti-vascular endothelial growth factor (VEGF) treatment with a specific monoclonal antibody (Bevacizumab) is beneficial in breast cancer when added to neoadjuvant chemotherapy but detrimental in an adjuvant setting [5,6]. In contrast, immunotherapy with checkpoint inhibitor antibodies such as anti-programmed cell death protein-1 (PD-1) (Nivolumab) and anti-cytotoxic T lymphocyte antigen-4 (CTLA-4) (Ipilimumab) seems to induce stronger anti-tumor immunity as post-surgical (adjuvant) therapy in patients with resectable metastatic melanoma and lung cancer when compared to neoadjuvant therapy; tumor mutational burden and T cell expansion were predictive of the anti-tumor response to checkpoint blockade [7,8]. In the context of immunotherapy, the microenvironmental heterogeneity of metastases presents an additional challenge and therapeutic obstacle. In colon or ovarian cancer, for instance, it has been shown that the immune environment can differ significantly between metastatic lesions in the same patient [9,10]. Therefore, more insights into the microenvironmental heterogeneity, for instance related to timing and location of metastases, are needed to determine the value of “one-size-fits-all” therapy versus more differentiated approaches [11]. This review will focus on tumor vasculature as a potential therapeutic target during metastatic tumor progression, and its potential role in enhancing immunotherapy.

## 2. Angiogenesis and Vessel Remodeling: Lessons Learned from Primary Tumors

Formation of new blood vessels from existing vessels is essential for tumor growth; this angiogenic process ensures oxygen and nutrient supplies and is in large part driven by growth factors such as VEGF [12]. However, the newly formed angiogenic vasculature is highly abnormal, and structurally and functionally different from vessels in non-cancerous tissues. During the angiogenic process, endothelial cells and pericytes, a mural cell type which stabilizes endothelial tubes, become hyper-proliferative, and pericyte coverage is partially or completely lost leading to substantial changes in the entire vascular bed [13,14]. Consequently, vascular integrity of angiogenic vessels is compromised and they become hyperpermeable; impaired blood perfusion causes low oxygen pressure (hypoxia) in the tumor microenvironment, immune suppression, therapy resistance and the propensity for metastatic dissemination [13]. The correlation between tumor perfusion, drug access and therapeutic outcome highlights the importance of a functional blood vessel network. Besides angiogenesis, tumors have developed other vascularization strategies such as vasculogenic mimicry and vessel co-option. Vasculogenic mimicry is a hallmark of aggressive tumors like melanoma, hepatocellular carcinoma, breast and ovarian cancers where tumor cells form vascular-like channels [15]. Histopathologically, vessel co-option has been observed in many primary cancers including lung, liver and brain and can occur in the presence or absence of angiogenesis. A feature of vessel co-option is that tumor cells grow alongside existing tissue blood vessels without inducing major structural modifications or new angiogenic vessels [16].

In recent years, the concept of normalizing tumor vasculature by reversing the chaotic angiogenic vasculature into a more orderly structure as a means of improving anti-cancer therapy, has gained momentum, strongly supported by preclinical data [17]. During vessel normalization, endothelial cells become less proliferative and barrier integrity is restored in part by upregulation of vascular adhesion molecules such as vascular endothelium (VE)-cadherin, and recruitment of pericytes [18]. Similar vascular normalizing effects are achieved by inducing pericyte quiescence and maturation along the vessel wall [19,20]. Much like vascular smooth muscle cells, pericytes can switch phenotype and exist in a synthetic (proliferative) or contractile (mature) state [21]. Whilst tumor pericytes are in a synthetic state, pericytes of normalized tumor vasculature display maturation and better alignment with endothelial cells [19].

Depletion of VEGF with low doses of a neutralizing antibody has been shown to induce vessel normalization by pruning of immature vessels and fortifying the remaining vessels with newly recruited pericytes [22]. A suite of alternative vessel remodeling strategies are currently being developed which involves targeting of vascular molecules such as Angiopoietins (Ang) and their tyrosine kinase receptors (Tie), specific VE-Cadherin targeting and vascular cytokine therapies [23]. For instance, antibodies which block the vessel de-stabilizing Tie2 receptor ligand Ang2 or dual anti-VEGF/Ang2 antibodies effectively increase endothelial cell integrity in mouse models of mammary carcinoma and pancreatic neuroendocrine tumors [24,25]. Various microRNAs and long-non-coding RNAs contribute to the angiogenic process as crucial regulators of VEGF signaling, hypoxia and endothelial barrier function; targeting these regulators opens opportunities for vascular normalization [18,26]. Further, peptide–cytokine fusion compounds which specifically bind to the abnormal tumor vasculature and deliver cytokines such as TNFα and the TNF family member LIGHT (or TNFSF14) normalize tumor vessels via endothelial cells or pericytes, respectively [19,27,28]. Although vessel normalization per se has no impact on primary tumor growth, improved vascular barrier function and tumor perfusion have been shown to reduce metastatic tumor dissemination in preclinical tumor models [29,30,31,32].

## 3. Vascular Hyperpermeability in the Pre-Metastatic Niche

Following escape from the primary tumor site, the pre-metastatic niche is the microenvironment in distant organs where circulating tumor cells lodge and extravasate to begin forming overt metastases. Hematogenous tumor dissemination is highly tissue tropic and exquisitely regulated by the primary tumor which uses long range mechanisms to prepare the ‘soil’ to capture and nurture circulating cancer cells (‘seeds’) in their host organs. For example, breast cancer frequently metastasizes to lung and bone, prostate cancer to bone, and colorectal cancer predominantly to liver [1]. The pre-metastatic niche is composed of hematopoietic cells, stromal cells and extracellular matrix (ECM) and its formation is a multistep process. First, primary tumor cells produce and secrete soluble factors and extracellular vesicles such as exosomes capable of acting at long distances. Second, these tumor-derived factors recruit bone marrow-derived and immune suppressor cells to secondary sites. Third, inflammation remodels the local microenvironment, most notably the vasculature and ECM. Circulating cancer cells are then captured and extravasate through the vasculature into tissue parenchyma, to begin forming metastatic nodules. Numerous tumor and stroma-derived soluble factors, often packaged in exosomes, bone marrow-derived suppressor and local stromal cells are important for the pre-metastatic niche formation. Exosomes play a crucial role in the development of pre-metastatic niches; as carriers of growth factors, lipids, DNA, mRNA and microRNAs they recruit inflammatory cells, induce angiogenesis and immune suppression, and also determine organotropism via specific exosomal integrins [33]. Factors which modulate the pre-metastatic niche have been extensively reviewed before [1,34,35,36]. Interestingly, vascular hyperpermeability and ECM alterations are amongst the earliest changes in the pre-metastatic niche [37,38,39]. For instance, well-known angiogenic modulators such as Ang2 and metalloproteinases such as MMPs 3 and 10 are upregulated and disrupt vascular integrity in the pre-metastatic lung of a mouse melanoma model [40]. In a metastatic breast cancer model, Gr-1^+^CD11b^+^ myeloid cells are recruited to the pre-metastatic lung and contribute to vascular remodeling by producing high levels of the angiogenic factor MMP9. Genetic deletion of MMP9 reduced lung metastases by normalizing the niche vasculature as evidenced by increasing endothelial cell-pericyte coverage and VE-cadherin expression [41]. Neuropeptide substance P secreted by circulating breast cancer cells induces upregulation of TNFα and Ang2 in brain endothelial cells which increases their permeability and transmigration of tumor cells into the brain [39]. Pericytes are also actively involved in the formation of the pre-metastatic niche. Lineage tracing revealed that pericytes at pre-metastatic sites display a highly proliferative and synthetic phenotype, resembling tumor pericytes, concomitant with upregulation of the pluripotent transcription factor Krüppel-like factor (KLF)4, which abrogates contractile marker expression [42]. In mice bearing metastatic colon cancer, compared to tumor-free mice, deposits of the ECM component fibronectin are enriched at the luminal side of liver blood vessels suggesting a causal relationship between fibronectin and capture of circulating cancer cells on the vessel wall. Indeed, cancer cells attach to fibronectin secreted by human umbilical vein endothelial cells (HUVEC) in vitro and form adherens junctions prior to transmigration [43]. Although our understanding of how the pre-metastatic niche is formed remains rudimentary, there are striking parallels between the pre-metastatic niche and the angiogenic environment in primary tumors indicative of angiogenic switching in host tissue prior to the arrival of tumor cells [44].

### Vessel Targeting and Remodeling in the Pre-Metastatic Niche

**Figure 1:**
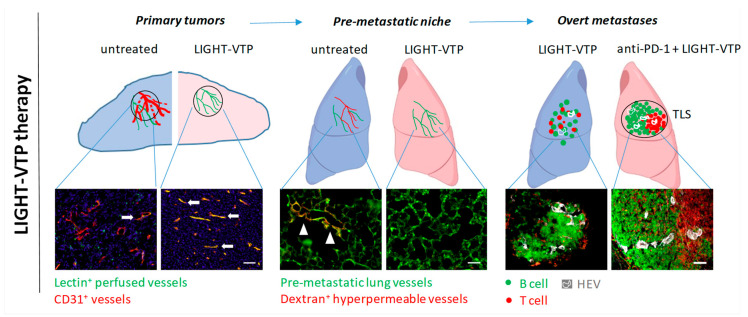
LIGHT-VTP therapy has distinct intra-tumoral effects at different stages during metastatic progression. Left, LIGHT-VTP therapy normalizes primary tumor vessels and improves vessel integrity and tumor perfusion, which also limits tumor cell intravasation into the circulation (30); arrows indicate well perfused vessels in yellow (overlay of CD31^+^ blood vessels (red) with i.v.-injected FITC-lectin (green) as surrogate marker for perfusion). Middle, LIGHT-VTP therapy reverses the hyperpermeability of pre-metastatic lung vessels (green) as measured by extravasated high molecular dextran (red). Arrow heads illustrate hyperpermeable areas in tumor-conditioned, untreated lung. In correlation with reduction of hyperpermeability, LIGHT-VTP treatment also reduces ECM (fibronectin) deposition and extravasation of circulating tumor cells (30). Right, in overt metastases, LIGHT-VTP therapy normalizes blood vessels, induces HEVs and small immature T and B cell clusters. LIGHT-VTP sensitizes to anti-PD-1 checkpoint inhibition and double treatment induces mature TLS with distinct T cell (red) and B cell (green) zones, and HEVs (white). Scale bars, 50 µm.

Vascular normalization at primary tumor sites can potentially reduce exosome release from cancer and endothelial cells, thus decreasing circulating pro-metastatic factors. Following surgical tumor removal, seed-soil interactions can also be therapeutically targeted to prevent metastasis formation. This includes interference with the niche immune milieu by neutralizing recruited inflammatory cells and angiogenic factors [44,45]. Furthermore, enhanced vascular ECM deposition in the pre-metastatic lung has been used to reduce lung metastases in a mouse model of primary bladder cancer. Circulating bladder tumor cells expressing the collagen receptor CD167a preferentially bind to airway smooth muscle cells which express high levels of collagen III (COLIII) and trigger Stat3 signaling in cancer cells. Napabucasin (BBI608) disrupts the COLIII–CD167a–Stat3 axis in the lung microenvironment and significantly reduces metastatic lung colonization [46]. Circulating cancer cells are also protected by platelets which play an essential role in tumor adhesion to lung endothelium. Inhibition of platelet cyclooxygenase (Cox-1), a key enzyme in prostanoids synthesis, reduces tumor-platelet aggregation, lung endothelium activation, and thus tumor cell seeding in the pre-metastatic niche; this may explain the anti-metastatic effects of the COX inhibitor Aspirin [47]. A new function-blocking antibody targeting the endothelial orphan receptor Tie1 (AB-Tie1-39) specifically suppresses tumor cell extravasation at secondary sites when applied in neoadjuvant or peri-operative settings, but is ineffective as adjuvant therapy. Pre-treatment of mice with AB-Tie1-39 but not IgG controls before intravenous (i.v.) injection of B16F10 melanoma cells reduces the number of metastatic lung lesions suggesting that Tie1 blockade prevents tumor cell lodging to the endothelium; this was confirmed on AB-Tie1-39 treated HUVEC cells in vitro. Mechanistically, Tie1 blockade specifically induced endothelial cell quiescence in the pulmonary pre-metastatic niche without changing the immune environment [29]. These findings also demonstrate the importance of timing in targeted therapy to coincide with biological events. Consistent with the notion that blood vessels in the pre-metastatic niche are angiogenic, the niche vasculature is sufficiently altered to enable specific targeting with small peptides initially discovered for their selective binding to angiogenic vessels in primary tumors using phage display libraries screens [48]. Lipid vesicles (micelles) coated with such tumor vascular targeting peptides (VTPs) specifically bind to pre-metastatic niches in lungs from Lewis lung carcinoma (LLC) bearing mice, but not to normal lungs in tumor naive mice. The compound LIGHT-VTP which delivers the cytokine LIGHT via a VTP to abnormal vessels is highly effective in targeting LLC or B16F10-induced pre-metastatic niches and ‘repairing’ vessel leakiness as demonstrated by reduced high molecular weight dextran extravasation, a readout for vascular hyperpermeability in tumor-conditioned lungs (Figure 1). Moreover, LIGHT-VTP pre-treatment reduces fibronectin deposition in lungs of cancer-bearing mice and prevents extravasation of intravenously injected circulating LLC cells through lung endothelium, thus preventing metastatic outgrowth [32]. These results suggest that the lung pre-metastatic niche vasculature can be normalized to suppress cancer seeding. Whether these early vascular changes can also be reversed at other common metastatic sites such as liver and brain needs to be determined [49].

## 4. Vascularization in Overt Metastatic Lesions

Following extravasation, cancer cells closely associate with micro-vessels in the host organ and subsequently induce all vascular changes essential for metastatic outgrowth [37]. In contrast to primary tumors, metastases can evolve in different spaces over time and metastasize to various secondary sites within the same individual; in addition, vascular and/or immune environments can differ between tumor lesions even in the same organ [9]. Understanding how metastatic tumors evolve and create their own microenvironment within distant organs will be crucial for developing effective combination therapies.

Liver, lung, and brain are amongst the most susceptible organs for metastases. Whilst angiogenesis is induced in the majority of metastatic cancers, alternative non-angiogenic strategies have also evolved such as co-option of the host tissue microcirculation similar to primary tumors [50,51]. Liver metastases of colorectal cancer, for instance, grow in distinct patterns which either preserve (replacement growth pattern) or destroy (desmoplastic and pushing growth patterns) liver parenchyma; angiogenic activity and the degree of vessel maturity as assessed by pericyte coverage varies significantly between the different growth patterns. Metastases that grow in a replacement pattern largely maintain the liver architecture and expand with minimal angiogenesis by co-opting existing liver sinusoidal blood vessels. In contrast, desmoplastic and pushing growth requires angiogenesis [50]. Similarly, in lung metastases of renal cell carcinoma, endothelial proliferation and microvascular density are lower when the normal lung parenchyma is preserved within the metastases [52]. In brain, clinical studies provide evidence for various degrees of vessel co-option in melanoma, breast or lung metastases [16]. In addition, the pattern of vascularization might be cancer type-dependent as shown in experimental brain metastasis models. For instance, in a mouse model of metastatic brain colonization after carotid artery injection and using multiphoton laser scanning microscopy to track cancer cells in relation to brain micro-vessels, human melanoma cells grow by vessel co-option, whereas human lung cancer cells initiate de novo angiogenesis [37]. Following direct intracranial injection most epithelial tumor cells co-opt native brain vessels; vascular cell proliferation is higher in tumors which do not grow invasively but instead form angiogenic nodules within the brain parenchyma [53]. Thus, in correlation with their growth pattern metastatic lesions can be heterogeneous with regards to endothelial cell proliferation, mural cell differentiation and endothelial coverage. Moreover, highly angiogenic primary tumors can give rise to mixed-phenotype or non-angiogenic metastases [52]. Vessel co-option seems to occur at higher frequency in metastases compared to primary tumors with >80% co-option found in a cohort of human lung metastasis from breast, colorectal and renal cancer [54], 30–40% in liver metastasis from colorectal cancers [55] and >90% in liver metastasis from brain cancer [56]. These vascular parameters will be important to inform future targeted anti-angiogenesis and immune therapies.

### Vessel Targeting and Remodeling in Metastases

The clinical management of metastatic disease remains challenging. The reasons for limitations in clinical efficacy of anti-angiogenesis therapy are unclear. Heterogeneous vascular compositions and growth patterns in advanced cancers are likely to play crucial roles [54,57]. For instance, in a spontaneous metastatic melanoma mouse model, two distinct tumor types with high and low angiogenic activity develop which also differ in vessel maturity and stabilization. Tumors harboring a mature vascular network develop resistance to anti-VEGF tyrosine kinase inhibition (PTK 787/ZK 222584 or PTK/ZK). Similarly, human melanoma metastases from relapse patients during Bevacizumab therapy display mature vessels with high pericyte coverage suggesting that more stable vessels are resistant to anti-VEGF therapy [57]. As in primary tumors, vessel co-option has been proposed to be one of the main reasons that metastases also have intrinsic resistance to anti-angiogenic therapy [51,54,58]. For example, growth of subcutaneously implanted 4T1 breast and C26 colorectal cancers is inhibited by anti-angiogenesis treatment with the tyrosine kinase inhibitor Sunitinib whereas experimental lung metastases of the same tumors are resistant to these growth inhibitory effects [54]. In addition to intrinsic resistance, vessel co-option may also cause acquired resistance to anti-angiogenic therapy in metastases similar to observations in primary cancer models [59,60]. In an experimental model of renal cancer metastatic to lung with mixed angiogenic phenotype, angiogenic metastatic nodules respond initially to Sunitinib but eventually develop therapy resistance coinciding with the switch from angiogenesis to vessel co-option [54]. Bevacizumab is clinically approved in conjunction with chemotherapy in human metastatic colorectal cancer. Nevertheless, liver metastases which progress under combination therapy display a high percentage of vessel co-option, and thus respond poorly to Bevacizumab [58]. Therefore, specifically targeting vessel co-option may be necessary in conjunction with anti-angiogenesis therapy. Cell motility and adhesion are crucial pathways for vessel co-option, and knockdown of the actin-related protein 2/3 complex (ARP 2/3) involved in actin-mediated cell motility, reduced vessel co-option and also increased responsiveness to anti-angiogenesis treatment in an orthotopic colorectal cancer liver metastasis model [58]. Similarly, knockdown of the neuronal cell adhesion molecule L1 (or L1CAM), utilized by brain metastatic cancer cells to grow along brain capillaries, decreases metastatic foci of lung cancer cells in brain [61]. These preclinical studies provide some interesting mechanistic insights into potential targeting of non-angiogenic metastatic outgrowth. An alternative strategy to systemic anti-vascular treatments is the use of targeting moieties to deliver therapeutic payloads to metastatic lesions. For instance, nanoparticles coated with 2 peptides specific for P selectin and αβ3 Integrin are able to home into different metastatic breast cancer lesions in the same individual which either express both or only one of the receptors; such approaches may be needed to deal with metastatic heterogeneity [62]. I.v. injection of TNFα conjugated to the angiogenic vessel-homing peptide ‘CNGRCG’ (NGR-TNF) reduces lung metastases in an adjuvant treatment regimen in mice bearing spontaneous breast cancer metastatic to lung, and in experimental melanoma metastases, demonstrating that blood vessels in metastatic lesions are sufficiently altered for targeted therapy [63]. Further, the VTP ‘CGKRK’ binds to 80% of blood vessels in experimental melanoma lung metastases and also blood vessels in human melanoma brain metastases. Moreover, four i.v. injections of the fusion compound LIGHT-VTP specifically normalize blood vessels within metastatic melanoma lesions as evidenced by reduced CD31^+^ vessel diameters and increased expression of contractile markers in pericytes, indicative of a more mature pericyte phenotype [32] (Figure 1). Thus, in preclinical models, the vasculature of overt metastases can be targeted and normalized similar to primary tumors.

## 5. Tumor Vessels and Immunotherapy: Lessons Learned from Primary Tumors

**Figure 2:**
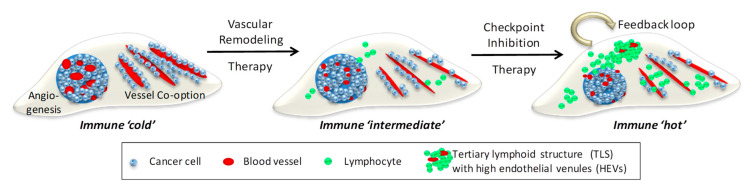
Combination Vascular-Immune Therapy in Metastatic Cancer. Left, depicted are 2 distinct metastatic vascularization patterns in host tissue, neo-angiogenesis and co-option of existing blood vessels. Developing tumors are devoid of lymphocytes and therefore immune ‘cold’. Middle, vascular remodeling therapy, e.g., anti-angiogenesis and anti-co-option strategies, normalize aberrant angiogenic vessels and reduce cancer adhesion to pre-existing vessels. Improved tumor perfusion facilitates lymphocyte influx and alleviates immune suppression, thus creating an immune ‘intermediate’ environment. Right, this sets the stage for checkpoint inhibitors to activate local adaptive immunity and generate a positive feedback loop where normalized vessels increase T cell infiltration and activated effector T cells normalize blood vessels for more lymphocyte influx. A critical mass of T and B cells forms clusters resembling tertiary lymphoid structures (TLS) containing high endothelial venules (HEVs) which further support naive and memory T cell migration. A self-perpetuating immune ‘hot’ environment has been created following sequential vascular remodeling and immune enhancing combination therapies.

Immunotherapy, in particular checkpoint inhibitors, have profoundly changed survival outcomes for some but not all cancers [64]. The angiogenic tumor vasculature plays an important role in regulating the response to cancer immunotherapy, in part by restricting T cell trafficking which results in immune ‘cold’ tumors [65]. In contrast, vessel normalization directly enables T cell influx into solid tumors, and indirectly changes immune suppression by reducing for example alternatively activated macrophages, myeloid suppressor cells and/or regulator T cells [66]. For instance, combined Ang2/VEGF inhibition with a bi-specific antibody promotes vascular regression whilst normalizing the remaining blood vessels in mouse models of breast and pancreatic neuroendocrine tumors. This in turn facilitates the extravasation of activated cytotoxic T lymphocytes and sensitizes tumors to anti-PD1 immune checkpoint therapy [25]. Synergism between vascular normalization treatment and checkpoint inhibitors have been shown in many preclinical models, and clinical trials of bi-specific Ang2/VEGF antibodies with immunotherapy in locally advanced or metastatic cancers are under way [23] (Figure 2). Importantly, low dose anti-angiogenic treatment regimens are more likely to improve immunotherapy than high doses which cause vessel death and exacerbate hypoxia [67]. In fact, vessel normalization and improved tumor perfusion are emerging as prognostic markers for enhanced responsiveness to immunotherapy [67,68,69]. Not only does vessel normalization improve lymphocyte influx, generation of IFNγ^+^ intra-tumoral CD4^+^ effector T cells following anti-PD-1/anti-CTLA-4 treatment in breast cancer models improves vessel function. Thus, effector T cells can normalize vessels in a positive feedback loop [68]. A scenario is conceivable where vessel remodeling therapy facilitates initial T cell influx and checkpoint inhibition generates effector T cells which in turn deepen vascular normalization and attract T cells in sufficient numbers to impact on tumor growth (Figure 2).

Furthermore, in human pancreatic tumors with favorable prognosis normalized vessels have been found in the vicinity of intra-tumoral high endothelial venules (HEVs) which are specialized post-capillary endothelial cells with typically ‘plump’ or cuboidal shape and essential for the de novo organization of tertiary lymphoid structures (TLS) in cancer [70]. HEVs normally reside in peripheral lymph nodes and regulate migration of naïve and memory T cells via expression of peripheral node addressins. When induced in tumors, they facilitate lymphocyte entry and formation of TLS which consist of HEVs, dense T and B cell zones and antigen presenting cells such as dendritic cells [71]. The presence of HEVs containing TLS as sites of immune priming in human cancers such as melanomas and sarcomas is generally indicative of better prognosis and responsiveness to checkpoint inhibitors [72,73,74]. Interestingly, recent preclinical studies suggest that tumor vessel normalization therapies can trigger TLS formation in the right inflammatory context. For instance, the compound LIGHT-VTP not only induces intra-tumoral vessel normalization and macrophage re-polarization, but also HEVs and TLS. These microenvironmental changes set the stage for successful combination treatment with anti-PD1/anti-CTLA4 checkpoint inhibitors in mouse models of pancreatic neuroendocrine and lung cancers [75]. Furthermore, LIGHT-VTP combined with low dose anti-VEGF treatment induces intra-tumoral HEVs in immune ‘cold’, therapy resistant mouse glioblastomas which in turn increases the responsiveness to anti-PD-L1 immunotherapy [20]. This is remarkable given the paucity of spontaneous TLS in brain cancer [76]. Therapeutic HEV/TLS induction in cancer lesions deserves further consideration when designing new immune combination treatments. Yet, much like vessel normalization in metastatic lesions our knowledge of HEV/TLS structures in metastatic cancer remains limited.

## 6. Immunotherapy in Metastatic Cancer

Metastatic lesions may have lower immunogenicity than the corresponding primary tumor and may also be systemically immune suppressive. For instance, a direct comparison of primary and metastatic human breast cancer demonstrated that metastases harbor less tumor infiltrating lymphocytes and chemotactic cytokines, express lower levels of PD-L1, and are therefore immunologically ‘colder’ [77]. Moreover, the presence of liver metastasis is associated with reduced response to anti-PD-1 checkpoint blockade (Pembrolizumab) in melanoma or non-small-cell lung cancer (NSCLC) patients compared to patients without liver metastasis [78]. Similarly, metastatic bone marrow lesions impair the efficacy of anti-PD-1 (Nivolumab) treatment in NSCLC patients compared to patients with cancer-free bone marrow [79]. Clinical data on the efficacy of checkpoint inhibition on metastases are still scarce since early clinical trials tended to exclude patients with metastatic lesions [80]. Nevertheless, more recent data are encouraging. For instance, NSCLC patients with brain metastases showed longer overall survival with anti-PD-1 checkpoint blockade (Pembrolizumab) but only in patients where PD-L1^+^ stromal and immune cells were detectable (in at least 1% of cells); tumor specimens from responders also expressed higher levels of granzyme B and proinflammatory chemokines such as CXCL9 and 10 compared to non-responders [81]. Nivolumab combined with Ipilimumab in melanoma patients with untreated brain metastases showed intracranial clinical benefits in 57% of patients [80]. It is possible that brain metastases originating from tumors with a high mutagenic load such as NSCLC and melanoma respond better to checkpoint therapy compared to metastatic tumors with less neoantigens.

Interestingly, in patients with melanoma brain metastases an increase in intracranial progression free survival was recorded using concurrent radiosurgery and checkpoint inhibition suggesting synergistic activities [82]. Radiation may increase release of antigens and/or cytokines at the metastatic site which enhances immunogenicity and response to immunotherapy. Additionally, microenvironmental changes may occur in remaining tissues after debulking surgery. For instance, in melanoma-bearing mice low dose radiation reprograms macrophages and attenuates angiogenesis which in turn normalizes blood vessels, facilitates T cell infiltration, and enhanced the efficacy of immunotherapy [83]. Given the prognostic value of tumor perfusion for immunotherapy in animal models [69], multiple clinical trials in metastatic cancer patients combining angiogenesis inhibition with immune checkpoint blockade are currently ongoing [84]. For instance, a phase 3 trial combining Prembrolizumab and Bevacizumab in patients with asymptomatic brain metastases from NSCLC and melanoma (ClinicalTrials.gov Identifier: NCT02681549) has begun. Early results from a trial combining Nivolumab with Bevacizumab in metastatic renal cell carcinoma are showing positive patient outcomes with increased CD8^+^ T cell infiltration and elevated IFN pathway gene expression in responders independent of PD-L1 status [85]. Few studies have assessed the presence of TLS specifically in metastatic cancer lesions compared to their well-established prognostic value in primary tumors [86]. However, in some colorectal liver metastases, functionally active TLS are found at the tumor-liver interface and predict better outcome following tumor resection [87]. Moreover, clusters of B lymphocytes, T cells, mature dendritic cells and HEVs are found in 25% of cutaneous melanoma metastases. Therein, re-arranged immunoglobulin genes in B cells are indicative of the formation of mature germinal centers and local antigen-driven B cell response further supporting that B cells play an important role in antigen presentation in TLS [72,88]. In metastatic breast cancer, TLS were found in lung and liver, but not in brain and ovaries; TLS positivity correlates with an increase in tumor-infiltrating lymphocytes and improved prognosis [76]. Overall, whilst TLS induction seems to be host tissue dependent, their prognostic value mirrors corresponding primary tumors [86]. Therefore, therapeutic induction of TLS in metastatic lesions may help to increase lymphocyte densities in sparsely infiltrated nodules and improve efficacy of checkpoint inhibitors. Indeed, in an experimental B16 melanoma model LIGHT-VTP therapy stabilizes/normalizes metastatic blood vessels and also induces TLS with dense HEV^+^ T and B cell zones. The presence of TLS sensitizes unresponsive lung melanoma metastases to anti-PD-1 blockade [32] (Figure 1). Whilst our understanding of the metastatic immune landscape is limited, there is sufficient evidence demonstrating that combination therapies which increase effector T/B cell densities and functionality will benefit patients with advanced cancer; these therapies may have to be tailored for the metastatic microenvironment.

## 7. Conclusions

Vascular remodeling is a hallmark of progressive cancer growth at primary and secondary sites. However, growth pattern and extent of vessel remodeling varies considerably depending on the primary cancer, host organ and state of tumor progression. In addition, organ-specific vascular beds contribute to microenvironmental heterogeneity [89]. There is also strong evidence that vessel status correlates with the tumor’s immunological landscape and response to immunotherapy. Once fully understood, the intricate relationship between blood vessels and immune cell trafficking during all phases of progressive tumor growth is likely to produce new multi-modal therapies [3]. Currently, the VEGF pathway is a major modulator of the tumor microenvironment. Curiously however, treatment delivers different outcomes at primary and metastatic sites. VEGF receptor tyrosine kinase inhibitors applied in adjuvant settings accelerated metastatic tumour growth [90], thus foreshadowing clinical results [5,6]. Whilst still poorly understood, alterations in the endothelial secretome and induction of a pro-inflammatory pre-metastatic niche following adjuvant anti-VEGF treatment may negatively affect tumor seeding in distant organs [90]. Nevertheless, VEGF/VEGF receptor inhibition by virtue of normalizing tumor vessels and alleviating innate immune suppression enhances intra-tumoral T cell function and may act in synergy with checkpoint inhibitors in primary and metastatic tumors [20,91]. Results from ongoing combination trials are greatly anticipated [84]. Based on our current knowledge, a new generation of drugs, combinations and timing regimens should aim to (i) induce long-lasting vessel normalization in primary cancers to improve tumor perfusion and reduce metastatic intravasation, (ii) repair vascular hyperpermeability at pre-metastatic sites to limit extravasation of circulating tumor cells, and (iii) normalize metastatic tumor vessels independent of their angiogenic or non-angiogenic growth pattern. Targeting angiogenesis and vessel co-option simultaneously in primary and metastatic tumor lesions is an interesting concept [58]. Using vessel normalization strategies as a basis to induce TLS and further enhance immune priming with checkpoint inhibition is another approach which has been shown in preclinical models to be likely effective at all stages of tumor progression [20,32,75]. To translate these findings and monitor vessel normalization efficacies at different sites, advanced imaging techniques will be required; clinically, assessing tumor oxygenation is in development for primary tumors such as breast and brain cancers using for instance optical imaging tools, PET and MRI [92,93,94]. Overall, tumor vessel activation, normalization, or differentiation is a prerequisite for effective immunotherapies in particular in immune ‘cold’ cancers and considering the microenvironment at numerous anatomical sites will improve patient outcomes.
